# Home is where the right ventricle lives: bronchopulmonary dysplasia-associated pulmonary hypertension and home ventilation

**DOI:** 10.1038/s41390-024-03550-4

**Published:** 2024-09-12

**Authors:** Samuel J. Gentle, Namasivayam Ambalavanan

**Affiliations:** 1 Department of Pediatrics, University of Alabama at Birmingham, Birmingham, AL USA; 2 Department of Pediatrics, Yale School of Medicine, New Haven, CT USA

## Commentary

In this month’s issue, Akangire et al. ^1^ characterize the clinical trajectory of bronchopulmonary dysplasia associated pulmonary hypertension (BPD-PH) in infants with ventilator-dependence at hospital discharge. As infants with BPD-PH are at increased risk for mortality^2^ and long term morbidity into adulthood,^3^ efforts to identify high risk subphenotypes^4^ are critical to improving outcomes in this population. Moreover, while inpatient risks of infants with BPD-PH have become increasingly described in recent years,^5^ ref. ^1^ further detail respiratory morbidity following discharge.

This observational study included infants discharged home on mechanical ventilation receiving outpatient care within the BPD Collaborative. Whereas a prior systematic review reported that 40% of infants with severe BPD develop BPD-PH,^5^ an even higher proportion of infants in this cohort of infants requiring home ventilation (~60%) were diagnosed with BPD-PH after 36 weeks’ PMA. This proportion of infants with tracheostomy impacted by BPD-PH approximates what has previously been reported in a single center study.^6^ This further substantiates the need for systematic screening for PH^7^ in infants with severe forms of BPD. Whereas outcomes related to the initial hospitalization differed between infants with and without BPD-PH including age at tracheostomy placement, transition to home ventilation, and discharge, the age at decannulation did not differ between groups. This inconsistency may result from the natural history of disease in infants with BPD-PH. Prior cohort studies have indicated that disease may resolve in a significant proportion of survivors by the age of three.^2,8^ This notably correlates with the postnatal age of decannulation observed in the present study in infants with pharmacologically treated pulmonary hypertension.

Of additional interest were the low and similar rates of mortality between infants with and without BPD-PH. Whereas this may be reassuring in an infant with BPD-PH who survives to discharge, the study design excluded infants with BPD-PH who died during the initial hospitalization who probably have more severe disease. Moreover, BPD-PH associated clinical severity may either postpone (as supported by the postnatal age at tracheostomy in infants with BPD-PH) or preclude tracheostomy placement. Taken in context to prior literature reporting the postnatal age of death in a more comprehensive population,^2^ this may suggest that there is limited incremental risk for BPD-PH associated mortality beyond the initial hospitalization. Other metrics of respiratory morbidity such as the number of respiratory subspecialty visits and readmissions following discharge also did not differ by BPD-PH status. This further suggests that the time period of most clinical concern is within the initial hospitalization, although the duration of initial hospitalization may be prolonged in surviving infants with severe BPD and BPD-PH.

In a single center study of 17 patients with overall mild subphenotypes of BPD-PH, there is some evidence that there may be improvement of echocardiographic BPD-PH severity following tracheostomy placement.^9^ In this study, echocardiographic markers including the tricuspid annular plane systolic excursion and maximal eccentricity index improved in echocardiograms subsequent to receiving a tracheostomy. However, more evidence of comparative approaches to tracheostomy placement in infants with BPD-PH are needed as these findings may more reflect the natural history of BPD-PH rather than tracheostomy mediated improvements in disease. Akangire et al. ^1^ only reported whether BPD-PH was pharmacologically managed and were otherwise limited in the ability to discern the epidemiology of BPD-PH following tracheostomy placement. Of interest is the higher frequency of diuretic usage in infants with BPD-PH compared to infants without BPD-PH. While there is limited evidence to support optimal pharmacologic approach to BPD-PH, growing consensus support for diuretic usage in this population^10^ and observational data suggesting right ventricular systolic improvement following diuretic exposure^11^ indicate the potential for benefit.

Among the limitations of the analysis are the ability to precisely discriminate infants with active BPD-PH from the remaining cohort. Disease status was defined as presence of PH at any time point beyond 36 weeks’ PMA. As 44% of the infants with BPD-PH were not discharged home on pharmacologic therapies, this subset of BPD-PH infants may not have even had active disease at the time of discharge. Moreover, it remains unclear whether efforts to stratify infants with BPD-PH by pharmacologic exposure sufficiently classifies the at-risk population with active disease. As a significant proportion of infants with BPD-PH have disease resolution in the interim between echocardiographic identification of disease and discharge (in this cohort around 12 months of age),^2^ additional discrimination of infants with active disease may be required to better define respiratory morbidity in infants with BPD-PH persistence at discharge.

This analysis further illuminates ongoing challenges in estimating clinical trajectories in infants with BPD-PH. Whereas one might predict that infants with both BPD-PH and underlying lung disease sufficient to warrant tracheostomy placement would be among the highest risk for mortality, this analysis did not reach that conclusion. Better biomarkers to aid clinicians in predicting disease severity in infants with BPD-PH are urgently needed, given the limited predictive utility of other plausible clinical covariates (e.g., need for tracheostomy placement).
